# How do executives’ synergistic allocation and organizational slack drive enterprise technological innovation?

**DOI:** 10.1371/journal.pone.0276022

**Published:** 2022-10-13

**Authors:** Guiyu Bai, Jing Zhao, Peng Xu

**Affiliations:** 1 Business School, University of Jinan, Jinan, China; 2 School of Business Administration, Shandong University of Finance and Economics, Jinan, China; 3 Center for Corporate Governance, Shandong University of Finance and Economics, Jinan, China; Sasin School of Management, Chulalongkorn University, THAILAND

## Abstract

Enterprise group is an important promoter to break the segmentation and achieve economies of scale. Technological innovation within the group is the key to improving market competitiveness, which has attracted common attention from academia and practitioners, but the decision-making mechanism of technology innovation in subsidiary is still needed. Based on the background of Chinese enterprises, through empirical analysis of panel data of 773 listed manufacturing companies for 5 consecutive years, we found: Parent-subsidiary executives’ synergistic allocation has a positive impact on the technological innovation of subsidiary; Parent-subsidiary executives’ synergistic allocation has a positive impact on the organizational slack of the subsidiary; The positive effect of executives’ synergistic allocation in parent-subsidiary corporations on the technological innovation of the subsidiary is realized by increasing organizational slack; Compared with private enterprise group, the positive influence of parent-subsidiary executives’ synergistic allocation on the technological innovation of subsidiary in state-owned enterprise groups is weaker; The longer the executive tenure is, the weaker the positive impact of organizational slack on technological innovation of subsidiary will be. On the one hand, this study enriches the theoretical research of technological innovation decision-making motivation; on the other hand, it provides empirical thinking for the improvement of parent-subsidiary executive collaborative governance mechanism and the improvement of governance efficiency.

## Introduction

Technological innovation is the engine of social development, the support of economic growth [1], and the crucial competitive advantage of enterprises. In the 2014 World Economic Forum, China proposed "innovation for all", which in turn radiates the nation with its national innovation strategy. With the development of globalization and knowledge economy, China emphasizes opening up to promote development, reform to promote innovation, cooperation to achieve win-win, and fully integration into the global innovation network. Technological innovation could help enterprises to cope with changes of external technology, industry environment, domestic and international economic situation. It is an important way to improve core competitiveness and promote sustainable development [2]. Enterprise group is a collection of parent companies and subsidiaries, which belongs to the intermediate organization between the market and enterprises and plays a leading role in economic development [3]. How to improve the level of technological innovation in the framework of the group, create an innovation level with distinctive advantages, and give full play to the role of the group in the integration of technology, capital and talent, has become the key to hatching high-quality innovative products and promoting the high-quality development of China’s economy.

Within the group framework, subsidiary is both passive executors and independent decision makers. The parent company can affect and supervise its subsidiary by regulations establishment, governance structure design and organizational culture constraints. Therefore, the effective balance of multiple interactions, the construction of a sound parent-subsidiary corporate governance system, and the play of intra-group synergy are crucial to the technological innovation of subsidiary [4]. At present, academic circles have carried out a series of researches on the technological innovation of subsidiary based on the particularity of the organizational form of enterprise groups. The Table 1 summarizes the more representative and diverse literature, which fully demonstrated the complexity of technological innovation decision-making of subsidiaries within the group framework. At the same time, although the existing research on technological innovation in enterprise groups is gradually more abundant, there are still some vacancies and the need for deep study.

**Table 1:** Comparative analysis of literature.

Author	Year	The main viewpoints	Research characteristics	Possible gaps
Efferin and Hopper	2007	The cultural control of parent company promotes the R&D collaboration between parent company and subsidiary company, thus improves the technical innovation performance of subsidiary company [5].	Take structural governance as the logic.	(1) Pay more attention to the overall operation mode of the group and lack the exploration of the specific governance mechanism.
Decreton et al.	2017	Subsidiaries have institutional dependence on their parent companies, and the closer cooperation within the group, the more conducive to technological innovation [6].	Emphasize the importance of group system.	
Ferraris et al.	2017	By analyzing the influencing factors and effects of open innovation of subsidiaries within the group framework, it is found that the internal network of the group has a stronger promoting effect on inward-oriented open innovation [7].	Different types of technological innovation.	
				(2) The discussion on improving the technological innovation path of the subsidiaries within the group is insufficient.
Xu et al.	2019	The parent shareholding has a negative impact on subsidiary’ responsive innovation of subsidiary, while the subsidiary with more managerial ownership choose relatively positive responsive innovation [8].		
Figueiredo et al.	2020	The knowledge transfer mechanism between parent and subsidiary company is conducive to the technology innovation of subsidiary, while the breadth and depth of subsidiary’ knowledge search will affect the relationship between them [9].	Understanding the driving force of enterprise technology innovation from the level of knowledge resources.	

Effective decision-making in innovation is decisive for innovation success [10, 11], therefore, paying attention to the process of enterprise innovation, the main body and influencing factors in the process of enterprise innovation are very important to the success of enterprise innovation, especially for large enterprise groups with complex organizational structure. On this basis, this paper takes listed companies in China as the research sample, introduces executives’ synergistic allocation in parent-subsidiary, and takes personnel management of executives as the research fulcrum to analyze the influence mechanism of this specific mechanism on subsidiary technology innovation. As an agent of the enterprise, executives are responsible for the development and daily operation of the enterprise and undertake the task of technological innovation. Executive efficiency directly affects the level of group innovation. Executives’ synergistic allocation in parent-subsidiary companies refers to the status of executives, including board of directors and managers, CEO duality combining the roles of CEO and chairman serving in both parent and subsidiary companies [12]. It is a governance mechanism for the group to achieve synergy on the unified, coordinated and centralized allocation of executives within the group [13]. Compared with previous studies, the possible contributions of this study are as follow: First of all, parent-subsidiary executives’ synergistic allocation reflects the structural arrangements made by parent companies to participate in subsidiary governance. This study examines the impact of parent-subsidiary executives’ synergistic allocation on subsidiary technological innovation and analyzes the mediating mechanism and moderating mechanism in the influenced path from the perspective of resource dependence, which further enriches the theoretical study of technological innovation decision-making motivation. Meanwhile, the relevant conclusions of this paper also further verify the positive value of executives’ synergistic allocation in alleviating parent-subsidiary information asymmetry [14] and promoting knowledge transfer, that provide empirical thinking for the improvement of parent-subsidiary executives’ synergistic allocation governance mechanism and the improvement of governance efficiency.

**Fig 1:**
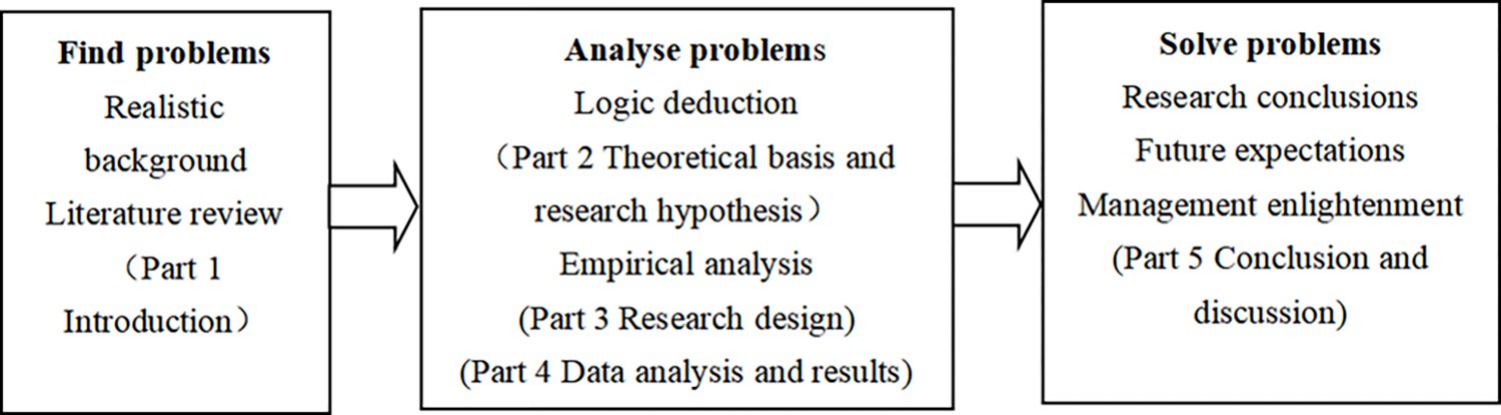
Research route.

Next, in the second part, this paper expounds the correlation between the parent-subsidiary executives’ synergistic allocation and the technological innovation of subsidiary, analyzes the intermediary role of organizational slack from the perspective of resource dependence, and explores the moderating effect of property rights nature and executive tenure. In the third part, the sample selection and data sources are introduced in detail, the variables are defined and measured, and the research models are designed. In the fourth part, this paper conducts descriptive statistical analysis and panel data regression analysis. The fifth part discusses the research results and significance, management implications and suggestions, and research deficiencies and prospects. The specific research route is shown below (Fig 1).

## Theoretical basis and research hypothesis

### The correlation between the parent-subsidiary executives’ synergistic allocation and the technological innovation of subsidiary

Compared with individual companies, subsidiary can also seek help from enterprise groups for technological innovation in addition to using their own resources. Innovation involves many factors in firm’s available resources. One of the paths for group value creation is to rationally allocate limited resources, guide attention and help subsidiary to deal with environmental changes and create new businesses [15]. However, under the parent-subsidiary corporate governance system, information asymmetry often affects the allocation of resources within the group and limit the support of parent company for technological innovation of subsidiary. On the one hand, the cross-organizational situation and internal-external differences between parent company and parent company prevent the parent company from knowing the strategic intention of the subsidiary [16]. It is difficult for parent companies to master all the information of subsidiary’ technological innovation and technological evolution, and there are cognitive constraints on professional knowledge, which cannot accurately predict the development prospects of innovation projects. Therefore, parent companies have a natural cautious attitude towards technological innovation activities with high task complexity and high investment risk [17]. On the other hand, as a spokesperson for subsidiary, the executive team affects the group’s decision-making. Few lobbying and performance hardly promote the group to support the technological innovation of the subsidiary [18]. In consequence, for subsidiary, the key to breaking through the technological innovation dilemma lies in gaining the support and trust of the parent company, as well as stimulating the initiative and enthusiasm of the senior management team of subsidiary [19].

Parent-subsidiary executives work together to establish a more direct link between parent-subsidiary companies, so that parent-subsidiary companies form a network outside the property relationship, which can reduce the degree of information asymmetry between parent-subsidiary companies and obtain more help from parent companies to promote technological innovation. Specifically, being a part-time executive with the will of the parent company can help the parent company understand more about the dynamics of subsidiary innovation activities and technology development, improve the visibility of subsidiary technological innovation, enhance the understanding and trust of the parent company in subsidiary technological innovation decision-making, and alleviate the adverse effects of inconsistent subsidiary with the parent company’s objectives and risk preferences under asymmetric information [20]. Subsequently, executives with dual identities will contact other executives, shareholders and institutional investors of the group company in the process of performing their duties, forming a personal relationship network within the group, and can use this network to obtain opportunities for subsidiary to cooperate with their parent companies. In the process of parent-subsidiary co-browsing the business environment and coordinating management matters, subsidiary frequently appear in the parent company’s attention range, which is easy to obtain the resources needed for innovation through strong relationships with parent companies across organizational boundaries. At the same time, parent-subsidiary executives’ synergistic allocation provides more possibilities for knowledge transfer between parent-subsidiary companies. Member enterprises within the framework of the group have a similar institutional environment and cultural atmosphere [21]. Parent-subsidiary executives’ synergistic allocation has capacity to increase effectively the communication frequency and emotional interaction between parent-subsidiary, thus forming a gradually strengthened sense of commitment may create favorable conditions for knowledge transfer, and better deliver detailed, implicit and integrated information [22]. In addition, this greatly enhance the willingness of parent company to share knowledge, provide more knowledge support for subsidiary technological innovation, and have a positive impact on subsidiary technological innovation. Based on the institutional theory and resource dependence theory, we argue that as parent-subsidiary executives’ synergistic allocation is immediately available and completely effective, and it provides a greater and safety knowledge sharing atmosphere and make executives’ positive impact on innovation.

Based on the above analysis, the following hypothesis is put forward:

H1: Parent-subsidiary executives’ synergistic allocation has a positive impact on the technological innovation of the subsidiary.

### Path analysis of the effect of parent-subsidiary executives’ synergistic allocation on subsidiary technological innovation from the perspective of resource dependence

Organizational slack refers to the accumulation of resources that exceed the minimum required investment in the production process of an organization [23], including knowledge, funds, products and personnel, which can be utilized and transformed by the organization to help the organization achieve its goals, besides buffer the internal-external pressures and environmental changes [24]. According to the theory of resource dependence, organizations not only need to adapt to the environment and supplement resources from the outside world, but also try to change the passive dependence status and control resources with their own advantages in the active face of the environment [25]. Consequently, although the subsidiary hopes to obtain the resource support of the parent company to promote technological innovation, it is more eager to accumulate resources and improve its own resource capacity to give play to the initiative of innovation. Executives’ synergistic allocation reinforces the adaptability and operational interaction between parent and subsidiary company, which enables subsidiary companies to further improve the possibility and potential of organizational learning on the basis of more and more updated resources and information. Through effective learning to expand their breadth and depth of knowledge, enterprises would increase the stock of knowledge. Simultaneously, executives’ synergistic allocation can encourage subsidiary management to respond rationally to resources. Under the trust and concern of parent company, the management of the subsidiary will not only make positive performance due to the attention and supervision, such as rational use of resources, reducing the pursuit of private interests, avoiding excessive expansion and excessive diversification, and gradually accumulate redundant resources to cope with the uncertainty of future development [26]. Moreover, they usually have richer management experience and higher level of management ability, to help subsidiary achieve technological synergy and scale effects, thereby accumulating financial capital, social capital, as well as information technology advantages, then increasing the stock of redundant resources [27].

Organizational slack resources have become the key support for subsidiary to implement technological innovation activities. Because technological innovation faces with many unknown difficulties and challenges, it is necessary to use sufficient funds, perfect equipment and rich technical knowledge to ensure that the goal can be achieved through constant trial and error [28]. Organizational slack can mitigate the impact of risks on organizations and form a relatively loose innovation environment within enterprises [29]. Subsidiary could use abundant resources to reduce time and economic cost, improve the resource integration ability of enterprise innovation, and ensure the innovation timeliness. Redundant resources can also be used to provide employees with high welfare and quality working environment to supplement innovation-related performance rewards, so as to mobilize the enthusiasm of employees to participate in technological innovation and strengthen the innovation motivation in various aspects. In addition, as organizational slack provides additional resources for enterprises, they can occupy an advantage in external competition, and it is easy to make use of these resources to attract relevant organizations for cooperation, create R&D alliances under the condition of resource complementarity, and improve the innovation level in the communication and learning environment [30]. For the management, abundant redundant resources can give managers more and more flexible rights of resource allocation, effectively relieve the resource pressure of managers in innovation decisions, which makes them more confident and determined to improve the success rate of innovation in the face of innovation problems [31].

From the above discussion, it can be seen that the parent-subsidiary executives’ synergistic allocation can reduce the degree of information asymmetry between the parent-subsidiary company, thereby building a strong sense of trust, and urging the parent company to input tangible and intangible resources to the subsidiary to form the organizational redundancy of the subsidiary. This is an important guarantee for the technological innovation of subsidiary, which will help them more calmly deal with risks and challenges in technological innovation and increase innovation vitality and motivation.

Based on the above analysis, the following hypotheses are put forward:

H2: Parent-subsidiary executives’ synergistic allocation has a positive impact on subsidiary organizational slack.

H3: Organizational slack has a positive impact on technological innovation of subsidiary.

H4: Organizational slack plays a mediating role in the relationship between parent-subsidiary executives’ synergistic allocation and subsidiary technological innovation, that is, the positive impact of parent-subsidiary executives’ synergistic allocation on subsidiary technological innovation is realized by enhancing subsidiary organizational redundancy.

### The moderating effect of parent-subsidiary executives’ synergistic allocation on the influencing path of the subsidiary’s technological innovation

#### The moderating effect of property rights nature

Under China’s special economic system, state-owned enterprises have dual attributes, which are not only the main body of market economic activities, but also an important tool for government participation and economic regulation [32]. They are quite different from private enterprises in their governance logic, decision-making mechanism and strategic goals. As the major shareholder of state-owned enterprises, the government entrusts SOES with more social responsibilities, and the decisions faced by senior executives are bound to be guided by government policies. They even have to comply with government governance goals and undertake non-economic goals, such as charitable donations and people’s livelihood services [33]. From the perspective of principal-agent, the owner of the state-owned enterprises are all the people, not a personal interests subject, thus forming a long chain of relationship between layers of principal-agent, and layer-by-layer agent lead to the "absence of ultimate control" and "insider control" [34]. So, the management has actual control which greatly increases the possibility that managers will squander resources for personal gain.

Therefore, the impact of parent-subsidiary executives’ synergistic allocation on organizational slack may change due to the particularity of property rights, which is embodied in the following aspects: Firstly, there are differences in the role of executives in resource accumulation and storage under different property rights situations. In general, executives’ synergistic allocation enhances the interference of concurrent executives in subsidiary governance and strategic decision-making, which facilitates them to control more resources. However, under the requirements of the government for policy objectives, concurrent executives may be more inclined to drive the management of subsidiary to use resources for social image management rather than accumulate redundant resources [35]. Secondly, the parent company’s supervision has certain constraints and variability. Compared with private enterprises, the complex agency problem of state-owned enterprises restricts the parent company’s supervision and control of subsidiary. When part-time executives obtain more relationships in the collaborative network, they are more likely to breed political motivation. Considering how they get political promotion during their tenure, they may even use resources that are not used by organizations to establish personal relationship networks. In order to meet their high-level demand for mutual relations and obtain support in a broader relationship to promote their own development [36], executives tend to reduce the accumulation of organizational redundancy.

Based on the above analysis, the following hypothesis is put forward:

H5: Property rights nature has a moderating effect on the relationship between parent-subsidiary executives’ synergistic allocation and subsidiary organizational slack. Specifically, compared with private enterprise groups, the parent-subsidiary executives’ synergistic allocation in state-owned enterprise groups has a weaker positive impact on the organizational slack of subsidiary.

#### The moderating effect of executive tenure

The upper echelons theory holds that managers’ values and background characteristics (age, profession, tenure, gender and experience) largely affect their decision-making tendency [37]. Executives usually make decisions based on their own understanding of the strategic situation they are in, which is accumulated during their tenure and reflects their management experience, psychological state and behavior mode [38]. Executives at different stages of tenure have different perceptions of the internal and external environment of the organization, which may adopt different management methods to solve business problems, thereby affecting the relationship between organizational slack and technological innovation.

Specifically, in the period of short tenure, managers show a proactive management attitude and open up in constant attempts and efforts because they want to quickly integrate into the enterprise and gain support and trust from all parties. In the short term, executives do not understand the intricacies within the company, and are more motivated to make strategic planning and decisions. In this context, executives may help companies identify opportunities faster and choose a better development path by improving production to demonstrate their talents or making improvements in organizational structure and employee management [39], which provides a good management environment for the positive impact of organizational slack on corporate technological innovation. On the contrary, with the gradual extension of tenure, managers running the same enterprise for a long time will reduce their interest and enthusiasm for work and tend to resist change and negative management [40]. In the meantime, considering that the enterprise has established a good relationship with stakeholders for a long time, the management team prefers to maintain stability and operate with a conservative attitude, rather than spend time and energy choosing new partners. This reduces the sensitivity of enterprises to external information, reduces cooperation channels, and weakens the positive impact of organizational slack on technological innovation.

Based on the above analysis, the following hypothesis is put forward:

H6: Executive tenure has a moderating effect on the relationship between organizational slack and the technological innovation of subsidiary. Specifically, the longer the executive tenure is, the weaker the positive impact of organizational slack on the technological innovation of subsidiary will be.

**Fig 2:**
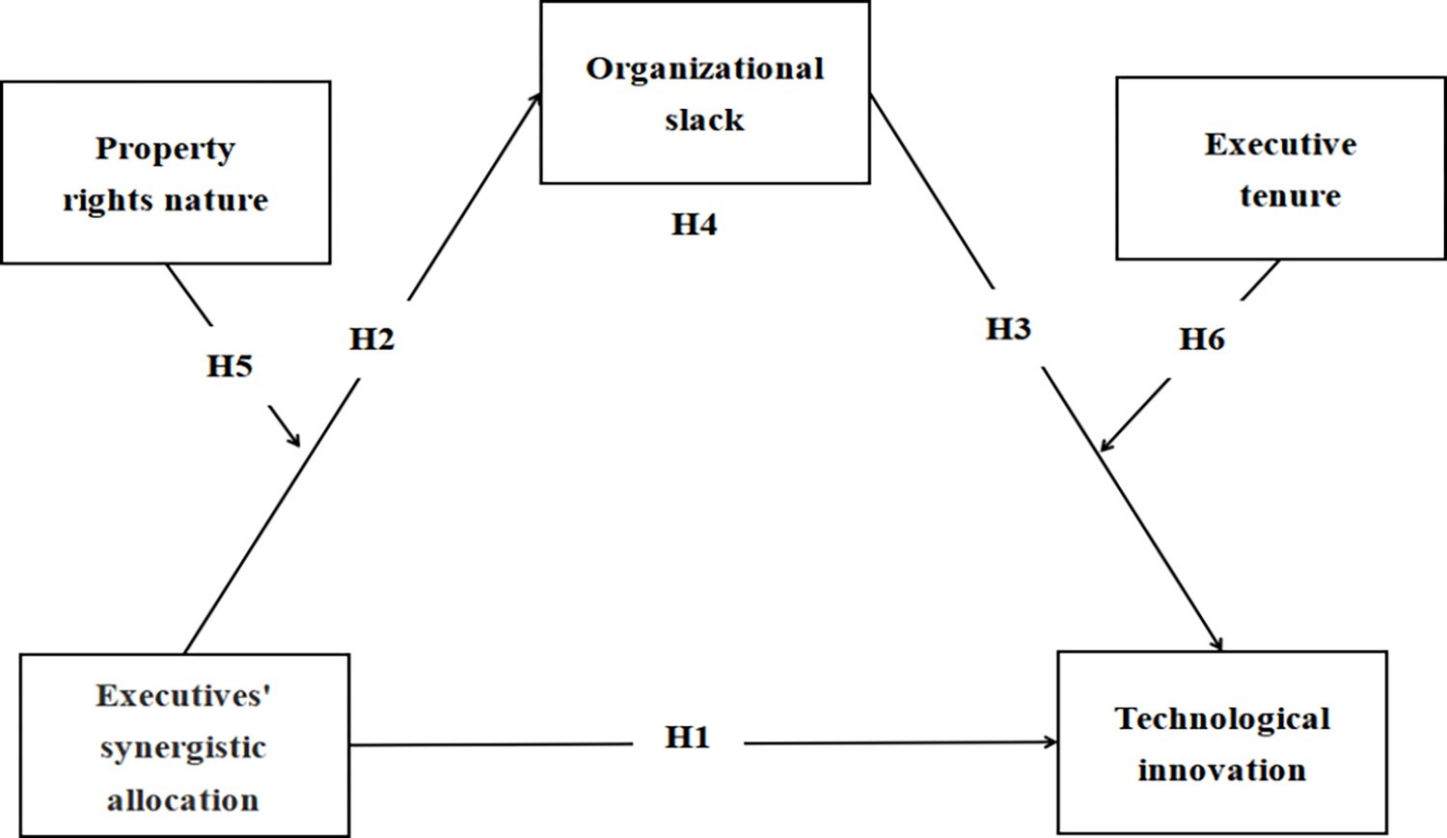
Hypothetical diagram.

To better understand the interplay between various factors: the parent-subsidiary executives’ synergistic allocation, executive tenure, property rights nature, organizational slack, we develop a research framework to test the combined effects of the above variables on technological innovation of subsidiary and also examine the contingent value of such connections (Fig 2).

## Research design

### Sample selection and data source

To test our hypotheses, we constructed a longitudinal data set of Chinese listed firms from the period between 2014 and 2018. The research objects of this paper are listed manufacturing companies belonging to enterprise groups. The data used in the empirical analysis are all secondary data, mainly from CSMAR database, which is the primary source of information on Chinese stock markets and the financial statements of China’s exchange-listed firms [41]. The sample and data collection steps are as follows: To begin with, based on the classification of group enterprises by Carney et al. (2009) [42], the authors make A preliminary selection of samples for all A-share listed companies in Shanghai and Shenzhen Stock Exchanges [43]. Subsequently, the sample observation interval is defined as 2014–2018, and the companies marked ST or stopped to be listed during the observation period are excluded. Finally, a data group composed of 773 listed companies for five consecutive years was obtained for empirical research.

### Definition and measurement of variables

#### Parent-subsidiary executives’ synergistic allocation (*ES*)

Referring to measurement method of the existing literature [44], the ratio of the number of subsidiary executives serving concurrently as senior executives in the parent company to the total number of subsidiary executives is used to measure the degree of executives’ synergistic allocation between parent and subsidiary companies. It should be noted that the scope of "executives" in this study is defined based on broad concepts, including board members, general managers, deputy general managers, financial officers, board secretaries and other managers stipulated in the company’s charter [45].

#### Technological innovation of subsidiary (*RD*)

The ratio of R&D investment to operating income is used to measure the support of enterprises for technological innovation. The higher the R&D index is, the higher-level technological innovation of enterprises will be [46, 47].

#### Subsidiary organizational slack (*OS*)

This paper selects the average of current ratio, asset-liability ratio and cost-income ratio as the measurement index of organizational slack [48]. Among them, current ratio = current assets/current liabilities, which is used to measure the ability of an enterprise’s current assets to be turned into cash to repay its liabilities before the maturity of short-term debts. The larger this index is, the more sufficient the resources to be utilized by an enterprise will be. Asset-liability ratio = total liabilities/total assets, which is used to measure the ability of an enterprise to use funds provided by creditors to carry out business activities. The larger the index is, the more funds an enterprise can provide for technological innovation will be. Cost-to-income ratio = (sales cost + management cost + financial cost)/operating income, which reflects the redundant resources integrated into the enterprise system [49].

#### Property rights nature (*PN*)

Set it as a virtual variable, state-owned enterprise group value is “1”, private enterprise group value is “0”.

#### Executive tenure (*LD*)

Referring to the previous common measurement methods [50], the average tenure of senior management team members, namely the existing tenure, is used as the measurement index of senior management tenure. By contrast, considering the executives may have multiple roles and there may be job switching, the article subtracted "the earliest date that a member worked at the company as an executive" from "the date when he or she last left as an executive" to obtain the number of days an executive held the job. In this way, it can better reflect the length of time that executives have been in the enterprise and judge the extent of their cognition and behavior modes affected by the enterprise. After that, the calculated data is processed logarithmically to eliminate heteroscedasticity to some extent.

Combined with existing studies, this paper also selects some variables that may affect the technology innovation of subsidiary as control variables. Including Company size (*Size*), Profitability (*ROE*), CEO change (*AL*), Board size (*BS*), Risk propensity (*Risk*), Pre-innovation performance (*PT*), Executive compensation (*LP*) and Executive age (*LA*). Since many current studies show that the relationship between innovation and firm performance is nonlinear, this study also introduces the square of *ROE*, an indicator of firm performance, into the model as a control variable to improve the overall accuracy of the model. The names and measurement methods of each variable are shown in Table 2.

**Table 2:** Definition and measurement of variables.

Variable name	Code	Index
Parent-subsidiary executives’ synergistic allocation	*ES*	The ratio of the number of subsidiary executives who hold concurrent positions as senior executives in the parent company to the total number of subsidiary executives
Technological innovation of subsidiary	*RD*	R&D as a percentage of revenue
Subsidiary organizational slack	*OS*	Average of current ratio, asset-liability ratio and expense-income ratio
Property Rights Nature	*PN*	Dummy variable, state-owned enterprise group is recorded as "1", private enterprise group is recorded as "0"
Company size	*Size*	The natural log of a company’s total assets at the end of the year
CEO change	*AL*	Dummy variable, marked as "1" if CEO change exists, marked as "0" if not.
Profitability	*ROE*	Corporate year-end return on equity = corporate net profit of the year/average balance of shareholders’ equity
Board Size	*BS*	Number of board members
Risk propensity	*Risk*	Long-term liabilities sum of items
Pre-innovation performance	*PT*	The number of patents filed in the previous year
Executive compensation	*LP*	The natural log of the average executive compensation
Executive age	*LA*	The natural logarithm of the average age of executives
Executive tenure	*LD*	The natural logarithm of the average executive tenure

### Research models

Combined with the research hypotheses, the regression models are designed as follows:

Model 1: $RD=c+\sum bjControl+a_{1}ES+\varepsilon$

Model 2: $OS=c+\sum bjControl+a_{1}ES+\varepsilon$

Model 3: $RD=c+\sum bjControl+a_{1}OS+\varepsilon$

Model 4: $RD=c+\sum bjControl+a_{1}OS+a_{2}ES+\varepsilon$

Model 5: $OS=c+\sum bjControl+a_{1}ES+a_{2}PN+a_{3}PN\times ES+\varepsilon$

Model 6: $RD=c+\sum bjControl+a_{1}OS+a_{2}LD+a_{3}LD\times OS+\varepsilon$

*Control* is the group of control variables, *c* is the intercept term, *ε* is the random disturbance term, *j* is the number of each control variable, *bj* is the regression coefficient of each control variable, and *a* is the regression coefficient of each explanatory variable.

Model 1 is a regression model of parent-subsidiary executives’ synergistic allocation on subsidiary technological innovation, which is used to test hypothesis 1. Model 2 is a regression model of parent-subsidiary executives’ synergistic allocation on subsidiary organizational slack, which is used to test hypothesis 2. Model 3 is the regression model of organizational slack on technological innovation of subsidiary, which is used to test hypothesis 3. Model 4 adds subsidiary organizational slack on the basis of model 1, which is used to test hypothesis 4, that is, the intermediary role of subsidiary organizational slack. On the basis of Model 2, Model 5 adds the product of property rights nature and executives’ synergistic allocation parent-subsidiary companies to test hypothesis 5 the moderating effect of the property rights nature of enterprise group. On the basis of Model 3, Model 6 adds the product term of executive tenure and subsidiary organizational slack to test hypothesis 6 the moderating effect of executive tenure.

## Data analysis and results

### Descriptive statistical analysis

Firstly, yearly descriptive statistics are conducted for the main variables to obtain the mean value, standard deviation, minimum value and maximum value of the variables. The results are shown in Table 3. From the variation trend and extreme value of standard deviation during the observation period, it can be seen that the application degree of parent-subsidiary executives’ synergistic allocation is different between enterprise groups, and the degree of differentiation tends to expand. The mean value, standard deviation and the difference between maximum and minimum value of the subsidiary’s technological innovation are all in an overall rising state. From the statistical results of organizational slack, it can be seen that the minimum value in five years is 0.289, indicating that organizational slack is prevalent in all sample enterprises. The mean value of property right is between 0.48 and 0.5, indicating that there is little difference between the sample number of state-owned enterprise groups and non-state-owned enterprise groups. In the data of executive tenure, the mean value decreases gradually from 8.536 to 8.354, indicating that the executive tenure in the sample enterprises has a decreasing trend.

**Table 3:** Results of descriptive statistical analysis.

Year	Variable	Mean	Standard deviation	Minimum	Maximum
2014	Parent-subsidiary executives’ synergistic allocation (*ES*)	0.174	0.110	0.000	0.583
	Technological innovation of subsidiary (*RD*)	3.446	2.839	0.003	23.99
	Subsidiary organizational slack (*OS*)	0.987	1.424	0.306	24.652
	Property Rights Nature (*PN*)	0.490	0.500	0.000	1.000
	Executive tenure (*LD*)	8.013	0.195	7.303	8.536
2015	Parent-subsidiary executives’ synergistic allocation (*ES*)	0.176	0.117	0.000	0.583
	Technological innovation of subsidiary (*RD*)	3.653	3.155	0.000	29.080
	Subsidiary organizational slack (*OS*)	0.899	0.779	0.308	12.046
	Property Rights Nature (*PN*)	0.488	0.500	0.000	1.000
	Executive tenure (*LD*)	7.926	0.209	7.171	8.564
2016	Parent-subsidiary executives’ synergistic allocation (*ES*)	0.174	0.122	0.000	0.583
	Technological innovation of subsidiary (*RD*)	3.763	3.652	0.000	46.72
	Subsidiary organizational slack (*OS*)	0.907	0.705	0.328	9.792
	Property Rights Nature (*PN*)	0.486	0.500	0.000	1.000
	Executive tenure (*LD*)	7.824	0.232	7.064	8.457
2017	Parent-subsidiary executives’ synergistic allocation (*ES*)	0.173	0.120	0.000	0.583
	Technological innovation of subsidiary (*RD*)	3.584	2.963	0.003	33.13
	Subsidiary organizational slack (*OS*)	0.910	0.889	0.312	15.024
	Property Rights Nature (*PN*)	0.484	0.500	0.000	1.000
	Executive tenure (*LD*)	7.704	0.254	6.894	8.448
2018	Parent-subsidiary executives’ synergistic allocation (ES)	0.170	0.119	0.000	0.632
	Technological innovation of subsidiary (*RD*)	3.927	3.938	0.000	58.820
	Subsidiary organizational slack (*OS*)	0.902	1.136	0.289	20.403
	Property Rights Nature (*PN*)	0.494	0.500	0.000	1.000
	Executive tenure (*LD*)	7.572	0.302	6.233	8.354

### Panel data regression analysis

According to the model designed above, regression analysis of panel data is carried out by STATA. Compared with the cross-section data and time series data, using panel data regression analysis is helpful to overcome the multicollinearity among variables, which can not only solve the endogenous from omitted variable problem, but also significantly reduce error term serial correlation with heteroscedasticity problems, to improve the accuracy effectiveness of the model and econometric estimation. The specific results of regression analysis are shown in Tables 4 and 5.

**Table 4:** Results of regression analysis—mediating effect.

Dependent Variable	*RD*	*OS*	*RD*	*RD*
Model	M1	M2	M3	M4
**Constant term**	-0.698	4.094***	-1.976	-2.022
	(-0.16)	(4.38)	(-0.45)	(-0.46)
**Control variables**				
*BS*	0.017	-0.004	0.018	0.018
	(0.51)	(-0.64)	(0.55)	(0.55)
*AL*	0.040	-0.011	0.035	0.044
	(0.70)	(-0.89)	(0.61)	(0.76)
*Size*	-0.146*	-0.125***	-0.100	-0.107
	(-1.66)	(-6.83)	(-1.13)	(-1.21)
*PT*	0.351*	-0.013	0.365*	0.356*
	(1.81)	(-0.31)	(1.89)	(1.84)
*ROE*	-2.141***	0.081	-2.137***	-2.163***
	(-7.55)	(1.35)	(-7.56)	(-7.65)
*ROE^2*	-1.082***	0.116	-1.092***	-1.121***
	(-2.78)	(1.43)	(-2.82)	(-2.89)
*Risk*	-0.117	0.031*	-0.124	-0.127
	(-1.46)	(1.90)	(-1.54)	(-1.59)
*LP*	0.394***	0.035*	0.371***	0.381***
	(4.25)	(1.77)	(4.02)	(4.12)
*LA*	1.480	-0.184	1.527	1.557
	(1.41)	(-0.83)	(1.46)	(1.49)
*LD*	-0.546***	-0.029	-0.515***	-0.539***
	(-4.42)	(-1.09)	(-4.21)	(-4.37)
**Independent variable**				
*ES*	0.709*	0.192**		0.645
	(1.77)	(2.27)		(1.61)
*OS*			0.335***	0.329***
			(3.57)	(3.50)
R^2^	0.052	0.026	0.056	0.057
F/Wald test	11.869***	6.039***	12.793***	11.951***
Hausman test	Fixed effect	Fixed effect	Fixed effect	Fixed effect
	(chi2 = 48.934	(chi2 = 58.419	(chi2 = 58.699	(chi2 = 60.143
	P = 0.000)	P = 0.000)	P = 0.000)	P = 0.000)

**Table 5:** Results of regression analysis—moderating effect.

Dependent Variable	*OS*	*RD*
Model	M5	M6
**Constant term**	3.876***	-9.202**
	(4.15)	(-2.00)
**Control variables**		
*BS*	0.001	0.018
	(0.08)	(0.56)
*AL*	-0.006	0.035
	(-0.46)	(0.60)
*Size*	-0.126***	-0.097
	(-6.83)	(-1.11)
*PT*	-0.009	0.384**
	(-0.22)	(2.00)
*ROE*	0.085	-2.107***
	(1.41)	(-7.50)
*ROE^2*	0.050	-1.152***
	(0.60)	(-2.98)
*Risk*	0.031*	-0.119
	(1.89)	(-1.49)
*LP*	0.034*	0.391***
	(1.73)	(4.24)
*LA*	-0.142	1.426
	(-0.64)	(1.37)
*LD*	-0.026	
	(-0.97)	
**Independent variable**		
*ES*	0.395***	
	(2.99)	
*OS*		9.281***
		(5.29)
**Moderator**		
*PN*	0.005	
	(0.07)	
*LD*		0.407*
		(1.87)
**Product term**		
*PN*×*ES*	-0.331**	
	(-1.97)	
*LD*×*OS*		-1.135***
		(-5.10)
R^2^	0.028	0.066
F/Wald test	5.422***	14.022***
Hausman test	Fixed effect	Fixed effect
	(chi2 = 59.815 P = 0.000)	(chi2 = 62.785 P = 0.000)

Note

***, **, * represents p < 0.01, p < 0.05, p < 0.1 respectively. The values of t are in parentheses. Hausman test criterion: When P is greater than 0.05, the original hypothesis is accepted, which means that the model is a random effect model. Otherwise, the original hypothesis is rejected, and the fixed effect model is adopted. Hausman set the model that cannot be distinguished by the test, using random effect model.

The P value of Hausman test in model 1 is less than 0.05, indicating that the fixed effects model should be used. While fixed effects model is valid and passes significance test (F = 11.869, R^2^ = 0.052), the regression coefficient of parent-subsidiary executives’ synergistic allocation as an explanatory variable is 0.709, and P<0.1, indicating that parent-subsidiary executives’ synergistic allocation has a significant positive impact on the technological innovation of the subsidiary. Hypothesis 1 is proved.

The P value of Hausman test in model 2 is 0.000, less than 0.05, indicating that the fixed effects model should be adopted. Fixed effects model is valid and passes the significance test (F = 6.039, R^2^ = 0.026). The regression coefficient of parent-subsidiary executives’ synergistic allocation as an explanatory variable is 0.192, and P<0.05, indicating that parent-subsidiary executives’ synergistic allocation has a significant positive impact on the organizational redundancy of subsidiary. Hypothesis 2 is proved.

The P value of Hausman test in Model 3 is 0.000, less than 0.05, suggesting that the fixed effects model should be adopted. And It is valid and passes the significance test (F = 12.793, R^2^ = 0.056). The regression coefficient of explanatory variable (organizational slack) is 0.335, and P<0.01, suggesting that organizational redundancy has a significant positive impact on the technological innovation of subsidiary. Hypothesis 3 is proved.

The P value of Hausman test in Model 4 still shows that the fixed effects model should be used. Fixed effects model is valid and passes the significance test (F = 11.951, R^2^ = 0.057). The regression coefficient of organizational slack of subsidiary company is 0.329 and P<0.01, while the regression coefficient of executives’ synergistic allocation is 0.645. Compared with the regression results of model 1, it is found that the regression coefficient of parent-subsidiary executives’ synergistic allocation does not pass the significance test when the mediating variable subsidiary organizational slack is added to the model, which indicates that subsidiary organizational redundancy plays a complete mediating role in the relationship between parent-subsidiary executives’ synergistic allocation and subsidiary technological innovation. Hypothesis 4 is verified.

**Fig 3:**
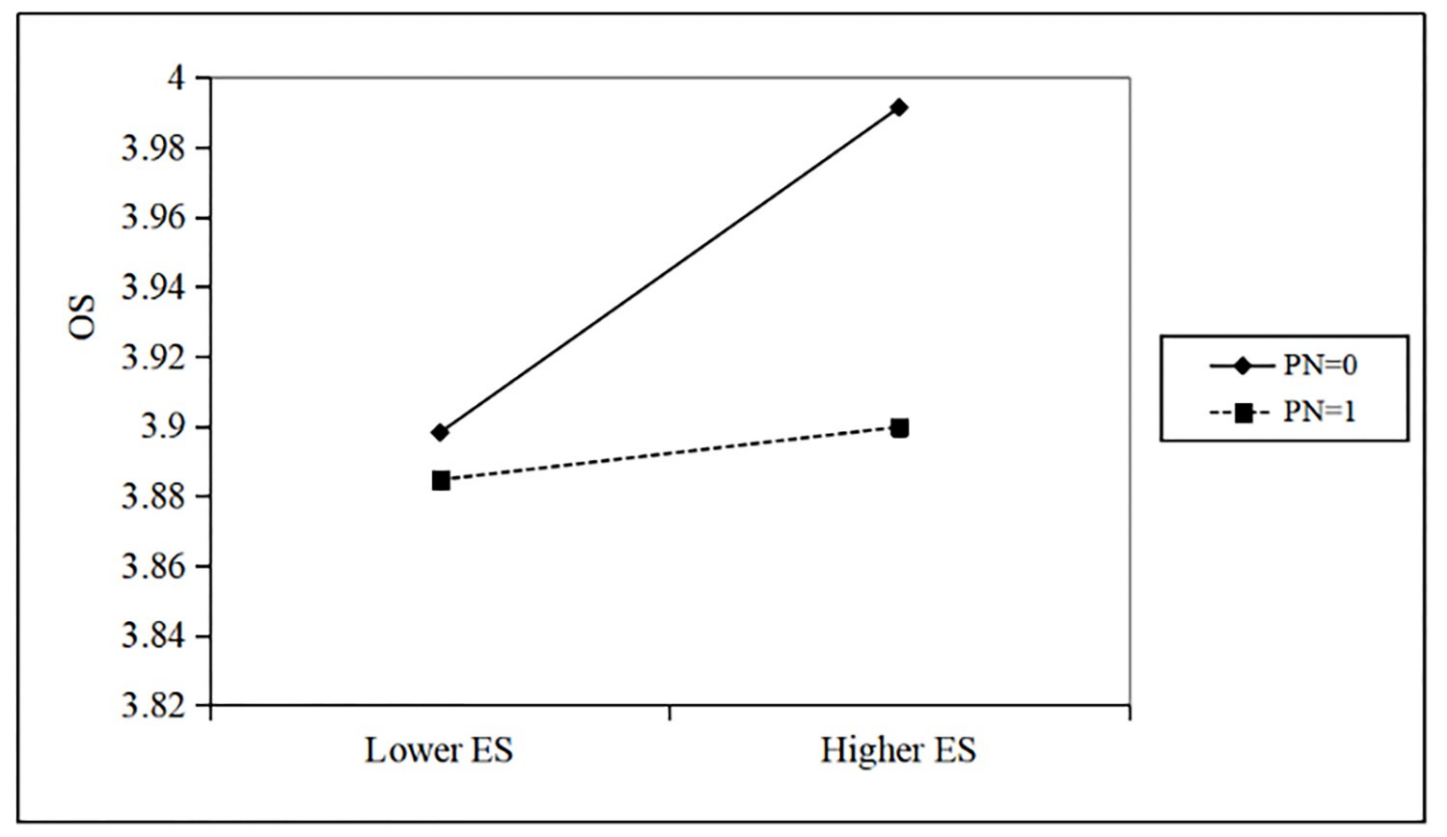
The moderating effect of property rights nature.

By observing the regression data of model 5, it is found that when the product of the property right of the regulating variable and executives’ synergistic allocation of the parent company and the explanatory variable are introduced into the model, the results of Hausman test still show that the fixed effects model should be adopted. The model is valid and passes the significance test (F = 5.422, R^2^ = 0.028). The regression coefficient of the product term is -0.331, P <0.05, which also passes the significance test. According to the test standard of regulating effect, if the product term is significant, it indicates that the regulating variable has a significant regulating effect in the main effect. Therefore, the regression data of this model verifies that property rights nature has a moderating role in the relationship between parent-subsidiary executives’ synergistic allocation and subsidiary organizational slack. That is, compared with private enterprise groups, the parent-subsidiary executives’ synergistic allocation in state-owned enterprise groups has a weaker positive impact on the organizational redundancy of subsidiary. Hypothesis 5 is verified. The moderating effect is shown in Fig 3.

**Fig 4:**
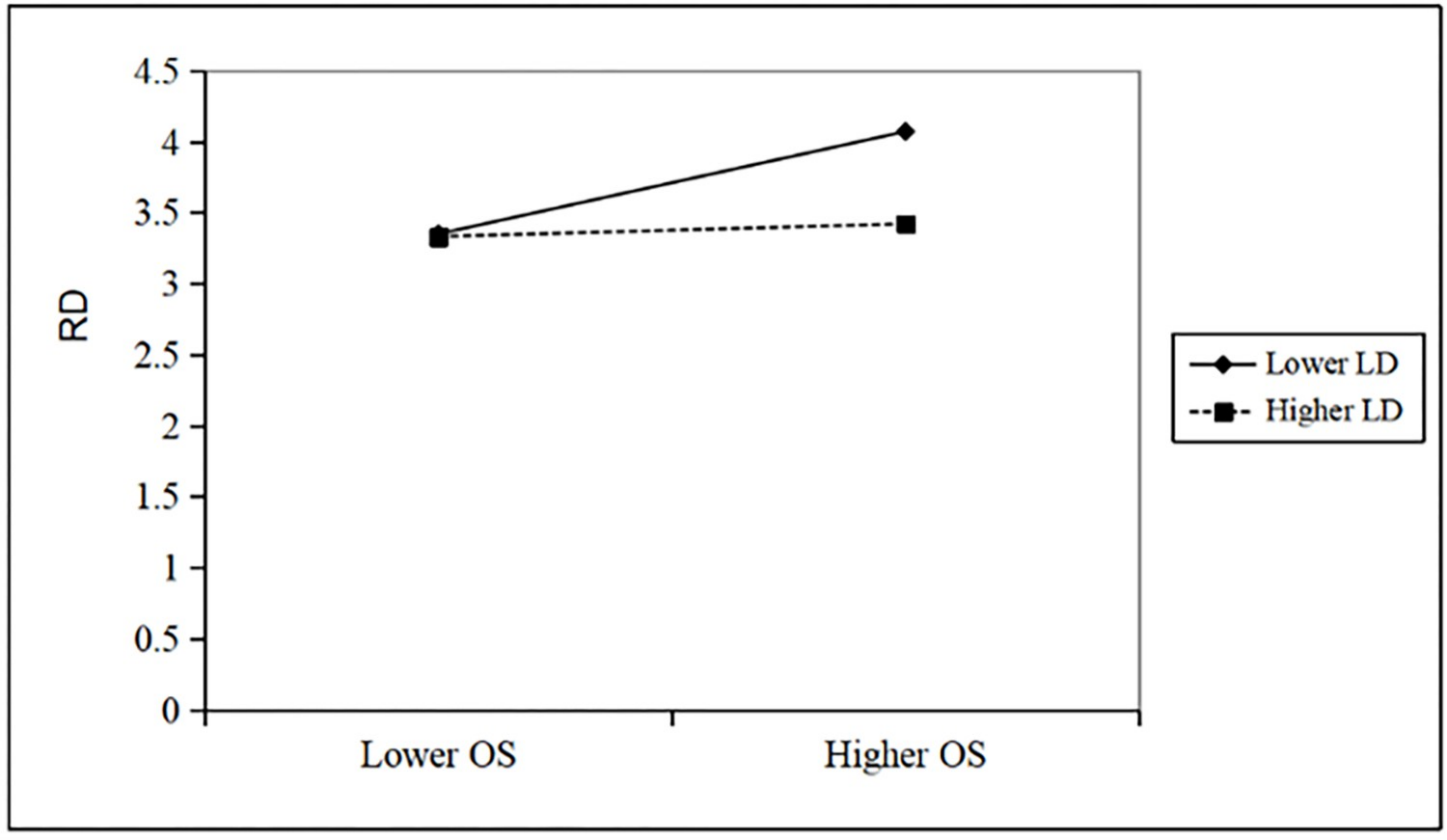
The moderating effect of executive tenure.

Similarly, when the product term of executive tenure and organizational slack of subsidiary is introduced into the model, the results of Hausman test in Model 6 still show that the fixed effects model should be adopted, and the model is valid and passes the significance test (F = 14.022, R^2^ = 0.066). The regression coefficient of product term is -1.135, p<0.01, indicating that the regulation effect is significant. In other words, the longer the tenure of senior executives is, the weaker the positive impact of organizational slack on the technological innovation of subsidiary will be. Hypothesis 6 is verified. The moderating effect is shown in Fig 4.

## Conclusion and discussion

### Study conclusions and significance

Technological innovation can give birth to new industries and new models, which is the source of enterprise groups to obtain and maintain long-term competitive advantages. It is necessary to adopt corresponding strategies and implement innovative decisions by executive teams. The responsibilities of executives within the group are complex. Some executives are responsible not only to the parent company, but also to the subsidiaries, thus affecting the development path of enterprise technological innovation. So, we examine how a parent-subsidiary executives’ synergistic allocation affects subsidiary technology innovation, and the moderating roles of executive tenure and the property rights nature. The main conclusions are as follows: Firstly, parent-subsidiary executives’ synergistic allocation has a positive impact on subsidiary technological innovation. Secondly, parent-subsidiary executives’ synergistic allocation can increase the subsidiary’s redundant resources. Thirdly, parent-subsidiary executives’ synergistic allocation promotes technological innovation of subsidiary by increasing organizational redundancy. Fourthly, compared with private enterprise groups, the positive impact of parent-subsidiary executives’ synergistic allocation on subsidiary organizational slack in state-owned enterprise groups is weaker. Fifthly, executive tenure has a moderating effect on the relationship between organizational slack and technological innovation of subsidiary. That is, the longer the executive tenure, the weaker the positive impact of organizational slack on technological innovation of subsidiary.

On the theoretical level, the existing research mostly discusses the relationship between the parent company and its impact on the technological innovation of the subsidiary from the perspective of the ownership of the parent company and the institutional inclination of the subsidiary but pays less attention to the role of senior executive allocation in the group. In this paper, the executives’ synergistic allocation in parent-subsidiary company, technological innovation and organizational slack are incorporated into the same framework, and the mechanism of action between variables is deeply demonstrated, which breaks the thinking limitations of existing research and enriches the theoretical model. Resource has always been a hot topic in management, many scholars are accustomed to applying resource dependence theory. However, this theory is mostly used to explain the relationship between enterprises and external markets, competition and cooperation between single enterprises, or the impact of internal initiatives. This paper analyzes group governance from the perspective of resource dependence, which broadens the application scope of the theory.

On the practical level, technological progress is the foundation of enterprises, technological innovation is crucial to all industries. Enterprise group is also a common form of senior organization in the current market economy. Thus, the focus of this paper on technological innovation in the group framework is a response to reality. The research conclusion could provide reference for the innovation problems of other companies or regions. Meanwhile, it verifies the positive effect of parent-subsidiary executives’ collaborative allocation on subsidiary technological innovation and provides a new path for group governance from the perspective of organizational slack. In the study, the nature of property rights, executive tenure and other contingency factors are considered, which makes the study more in line with the practice.

### Managerial implications and suggestions

To begin with, in practice, when the parent-subsidiary corporate governance mechanism is designed, it is necessary to attach importance to the positive governance role of executives’ synergistic allocation and make use of dual-status executives to realize intra-group collaboration, so as to change the phenomenon of "gathering but not group". Executives’ synergistic allocation provides a more direct channel for parent-subsidiary interaction. Out of trust, the parent company will give its subsidiary certain resources to carry out technological innovation. A good relationship between the parent company and the subsidiary company can enhance the overall cohesion and scale effect of the group. Hence, (1) For the subsidiary company, it should take the initiative to strengthen the communication with the parent company by utilizing the executives’ synergistic allocation, establish the intangible close advantage, and improve the enterprise creativity on the basis of mutual attention and understanding. (2) Serving as a bridge between executives is the key to the entire governance mechanism. The group headquarters should enhance the management and supervision of senior executives, set specific performance appraisal indicators and incentive plans, and avoid damaging the interests of the group due to personal interests, especially the accumulation of personal resources through the association network. (3) Enterprise groups with different property rights should implement differentiated parent-subsidiary executives’ synergistic allocation and fully consider the particularity of state-owned enterprise groups under China’s special economic system.

Moreover, technological innovation is the result of enterprises’ pursuit of efficiency and continuous adaptation to economic development. Faced with risks and uncertainties, subsidiary need to obtain resource support from the parent company, so the group headquarters should have a comprehensive understanding of the value and importance of technological innovation. (1) Resources are the water of life for enterprise technological innovation, which can give innovation vitality and help enterprises in life-or-death situations and crises. In order to ensure the smooth progress of the subsidiary’s technological innovation, the parent company should allocate resources scientifically and reasonably to create superior resource conditions, including capital, personnel and knowledge, for the subsidiary. (2) Executive tenure is an important factor affecting executive attitude. A negative attitude will consume enterprise resources and reduce innovation efficiency. Accordingly, enterprises should do a good job in executive deployment and internal control, formulate reasonable executive tenure, and optimize team composition to improve management performance.

### Research insufficiency and prospect

1. This paper only takes listed manufacturing companies in China as the research sample, without involving other countries and non-listed companies, so that the sample size is limited, and more extensive exploration can be carried out in the future. At the same time, subsidiaries in different life cycles have different dependence on the group, and the demand for resources and technological innovation is also different. In the future, we may study how the parent-subsidiary executive synergy affects the technological innovation of subsidiaries in different stages of development, growth, maturity and recession.
2. The technological innovation mentioned in this paper refers to the overall level of innovation input within the enterprise. It does not further distinguish the types of technological innovation and the differentiated impact of parent-subsidiary executive collaboration on technological innovation. In the future, we can try to study green innovation, substantive innovation and strategic innovation.
3. The collaborative configuration of parent-subsidiary company executives defined in this paper is a concept in a broad sense. In reality, there are many situations such as financial collaboration, business collaboration, technology collaboration and so on, and there are also differences in the position of senior executive, which can be refined from this perspective later.
